# Patterns and determinants of malaria risk in urban and peri-urban areas of Blantyre, Malawi

**DOI:** 10.1186/s12936-016-1623-9

**Published:** 2016-12-08

**Authors:** Don P. Mathanga, Atupele Kapito Tembo, Themba Mzilahowa, Andy Bauleni, Kondwani Mtimaukenena, Terrie E. Taylor, Clarissa Valim, Edward D. Walker, Mark L. Wilson

**Affiliations:** 1 Malaria Alert Centre, College of Medicine, University of Malawi, Blantyre, Malawi; 2 Department of Osteopathic Medical Specialties, College of Osteopathic Medicine, Michigan State University, East Lansing, MI USA; 3 Department of Immunology and Infectious Diseases, Harvard School of Public Health, Boston, MA USA; 4 Department of Microbiology and Molecular Genetics, Michigan State University, East Lansing, MI USA; 5 Department of Epidemiology, School of Public Health, University of Michigan, Ann Arbor, MI USA

**Keywords:** Malaria epidemiology, Urban health facilities, Peri-urban, Rural travel, Malaria disease risk, *Anopheles* vectors, Malawi

## Abstract

**Background:**

Although malaria disease in urban and peri-urban areas of sub-Saharan Africa is a growing concern, the epidemiologic patterns and drivers of transmission in these settings remain poorly understood. Factors associated with variation in malaria risk in urban and peri-urban areas were evaluated in this study.

**Methods:**

A health facility-based, age and location-matched, case–control study of children 6–59 months of age was conducted in four urban and two peri-urban health facilities (HF) of Blantyre city, Malawi. Children with fever who sought care from the same HF were tested for malaria parasites by microscopy and PCR. Those testing positive or negative on both were defined as malaria cases or controls, respectively.

**Results:**

A total of 187 cases and 286 controls were studied. In univariate analyses, higher level of education, possession of TV, and electricity in the house were negatively associated with malaria illness; these associations were similar in urban and peri-urban zones. Having travelled in the month before testing was strongly associated with clinical malaria, but only for participants living in the urban zones (OR = 5.1; 95% CI = 1.62, 15.8). Use of long-lasting insecticide nets (LLINs) the previous night was not associated with protection from malaria disease in any setting. In multivariate analyses, electricity in the house, travel within the previous month, and a higher level of education were all associated with decreased odds of malaria disease. Only a limited number of *Anopheles* mosquitoes were found by aspiration inside the households in the peri-urban areas, and none was collected from the urban households.

**Conclusion:**

Travel was the main factor influencing the incidence of malaria illness among residents of urban Blantyre compared with peri-urban areas. Identification and understanding of key mobile demographic groups, their behaviours, and the pattern of parasite dispersal is critical to the design of more targeted interventions for the urban setting.

**Electronic supplementary material:**

The online version of this article (doi:10.1186/s12936-016-1623-9) contains supplementary material, which is available to authorized users.

## Background

Urban and peri-urban areas are considered to be at lower risk of malaria compared to more rural areas because of improved housing, higher socioeconomic status and limited number of breeding sites [1]. However, since the number of urban malaria cases account for 6–28% of the estimated global annual malaria disease incidence [2], urban malaria remains a concern in the context of elimination efforts. This is complicated by reports that in some cities, malaria illness and vector breeding sites are associated with urban agricultural practices [3, 4]—practices that are quite common in African cities. Rapid urbanization has sometimes meant that in the periphery of cities, uncontrolled commercial activities create environments that are conducive for *Anopheles* vector breeding, perhaps explaining why transmission is more intense in such settings. Therefore, to better address the problem of malaria in urban settings, there is a need to understand epidemiologic patterns of transmission and disease in urban/peri-urban contexts and how this is different in rural settings.

Blantyre city in Malawi is experiencing one of the highest urbanization rates in Africa [5]. A few studies have focused on malaria risk within Blantyre city [6, 7], but investigations comparing malaria incidence within the city limits with that of peri-urban and rural contexts are rare [8]. In this report, risk factors associated with malaria were evaluated in both urban and peri-urban areas to better understand the variation of malaria risk across these settings. Findings from this study highlight factors that drive urban and peri-urban malaria burden, and identify high-risk populations, which in turn should inform efficient and targeted interventions for this population.

## Methods

### Study setting

This study was conducted in Blantyre city (15°47′05″ S; 35°00′30″ E), the commercial capital of Malawi, with a population of ~1,100,000 [9]. Blantyre is located in the Shire highlands of southern Malawi (~800 to 1600 m elevation), and has one of the highest annual population growth rates (5.2%) in the world, with the majority of city residents living under poor, overcrowded and sub-standard housing conditions [5]. Health care, both curative and preventive, is provided at no cost through a network of 15 public clinics and two hospitals. Malaria control efforts, based on the premise that there is active urban transmission, are guided by three main strategic activities: prompt access to effective anti-malarial drugs, scale-up of long-lasting insecticide-treated nets (LLINs) and use of intermittent preventive treatment (IPT) by pregnant women. In July 2012, a mass distribution campaign of Olyset® nets (Sumitomo Chemical Co., Japan) was carried out in the city, during which one LLIN was distributed for every two people per household. Surveys conducted in the study area during the study period indicated that 84.2% of the households owned at least an LLIN and 67.3% of individuals (of all age groups) had slept under an LLIN the night before the survey [8].

### Study design

**Fig. 1:**
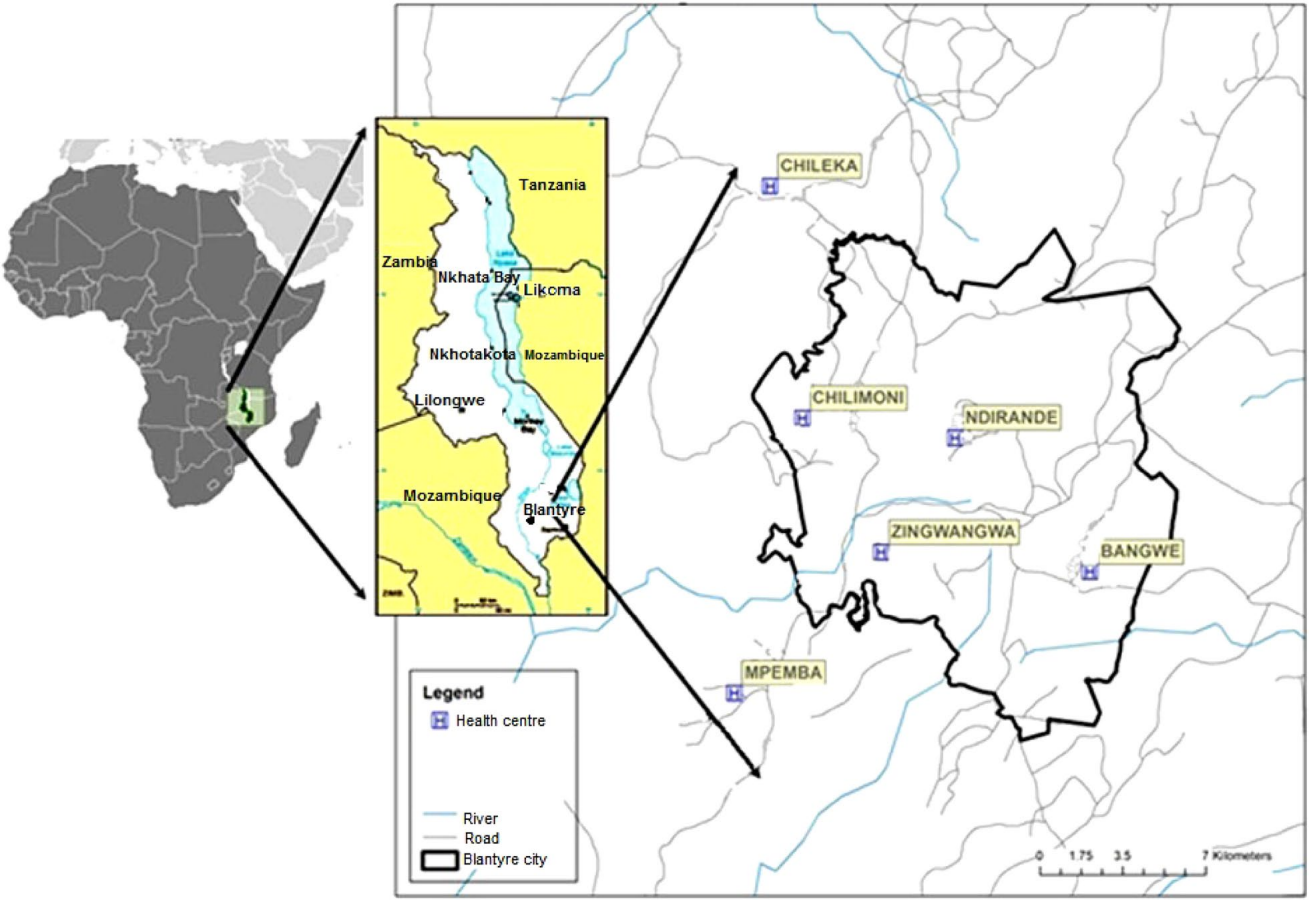
Map of Blantyre city, Malawi showing locations of the four urban and two peri-urban health facilities

This prospective, health facility-based, matched, case–control study recruited cases and controls between April 2012 and October 2015 from six health centres: four located within the government-defined city limits were considered ‘urban’ (Bangwe, Chilomoni, Ndirande, Zingwangwa) and two just outside the city limits were considered ‘peri-urban’ (Chileka, Mpemba) (Fig. 1). To be eligible to participate, children had to be aged 6–59 months, attending a sick clinic and living in the study area at the time of recruitment. Cases were defined as children who had malaria illness by meeting three criteria: either an axillary temperature ≥37.5 °C or history of fever (≤48 h), a positive blood smear by microscopy for *Plasmodium* infection (any parasite density), and a positive PCR test for *Plasmodium falciparum*. Controls were defined as children of the same age group who came to the same sick clinic with or without fever and had a negative blood smear and negative PCR test. Cases were individually-matched to two controls by age category (6–24 or 25–59 months), zone of residence within the HF catchment area, and time of diagnosis (within 4 weeks).

### Blood sample testing

Thick blood smears, whose results were used to provide standard of care, from sick children were stained with Giemsa at the HF and examined for *P. falciparum* by an experienced laboratory technician who was blinded to clinical symptoms of the study participants or reason for the visit. Parasite density was calculated by counting the number of asexual parasites per 200 white blood cells, and assuming a white blood cell count of 8000/μL of blood. Slides were considered negative if no parasites were found after examining 100 high-power fields. To ensure correct classification of participants as cases and controls, dried blood spots on filter-paper samples were subjected to a standard, species-specific, nested quantitative polymerase chain reaction (qPCR) assay for the detection of *P. falciparum* at the University of Malawi College of Medicine Molecular and Genomics Core Laboratory.

For each participant, a home visit was scheduled within four weeks of recruitment that involved a structured interview, direct observations of the household environment, and indoor aspiration of resting mosquitoes. The interview of each participant’s parent or guardian sought demographic information, measures of socio-economic status, malaria knowledge, attitude and practice, recent travel and overnight sleeping history, LLIN use, and other variables hypothesized to affect household level risk. Recent travel and sleeping away was defined as spending a night away from the house, and specifically outside of the city limits for those whose residence was inside the city limits. For those in the peri-urban area, travel and sleeping away was defined as spending a night sleeping away from the house in a place other than within the Blantyre city limits.

Direct observations of the household environment were made to assess house construction (wall type, roof type), number of windows, presence/absence of screens, open or closed eaves and potential mosquito breeding sites within a 20-m radius of the household. After the interview and direct observation, a battery-powered aspirator was used to collect resting mosquitoes from the inside of the primary dwelling.

### Ethical review

Written informed consent was obtained from parents or caregivers of each study participant prior to enrolment in the study. The Institutional Review Boards of the College of Medicine University of Malawi, University of Michigan and Michigan State University reviewed and approved the entire protocol.

### Statistical analysis

To assess the importance of travelling and socio-economic variables in the urban and peri-urban setting, odds ratios, Wald confidence interval and P values were estimated through conditional logistic regression to adjust for the matching factors. Since age group, zones where participants lived, and season were all part of the matching strata, associations between those variables and the probability of clinical malaria were not reported. Hypotheses related to differences in risk factors in the urban and peri-urban setting were tested by including interaction terms between each variable and an indicator variable defined as ‘urban’ or ‘peri-urban’, depending on the HF that the child visited (urban HFs were Bangwe, Ndirande, Zingwangwa, and Chilomoni; peri-urbanHFs were Chileka and Mpemba). This was done without the main effect for urban/peri-urban, since that was already accounted for in the matching factor. Given the correlation among variables representing house ownership, house construction, and sanitary system, summary scores based on a principal component analysis (PCA) were created to study association between these variables and clinical malaria in regressions. When creating the principal component scores, a model including only variables about house ownership, house construction, sanitary system, and one including those variables and possession of household items (radio, bike, car, TV) and electricity was evaluated. A more parsimonious model was chosen, since the model including possession of household items and electricity explained a lower percent of the variability in the first components. Possession of household items and electricity in the house, as appropriate, were considered separately in regression models. Components that explained 75% of variability in the data were selected and used in regressions. Each component was interpreted and named based on those variables that had loadings greater than the absolute value of 0.40. Multiple regressions models were selected based on best sub set regression in which variables were kept in models if P values were <0.10. All tests were considered statistically significant to an alpha-level of 0.05. All analyses were performed using SAS v9.4.

## Results

### Population description

**Fig. 2:**
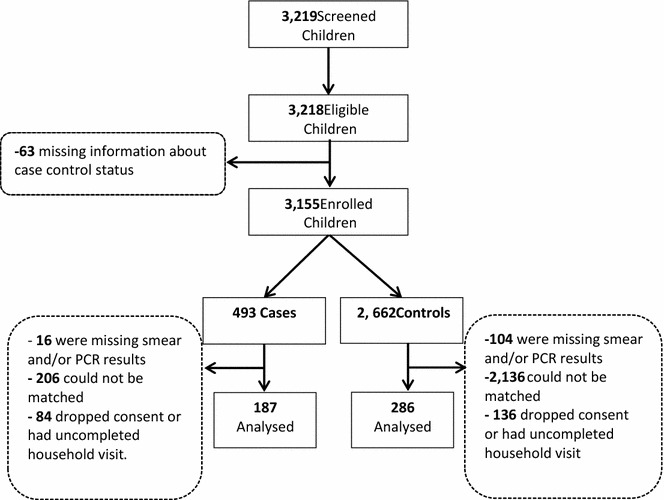
Number of children who were initially screened, enrolled, and excluded or included in the analysis by status as either cases or controls

A total of 3155 children were enrolled in this study and 473 were analysed (187 cases and 286 controls) after excluding 2432 children that could not be matched, 120 with unconfirmed diagnosis and 231 that dropped consent or whose houses could not be located (Fig. 2). Most study drop-outs occurred among children living in the urban setting, resulting in a total of 274 and 179 analysed from urban and peri-urban HFs, respectively, with 56% of cases and 61% of controls having attended peri-urban HFs (Table 1). The mean, median and 90th percentile time to the identification of a control for every case was 2.1, 1–7 days respectively.

**Table 1:** Distributions of enrolled and studied malaria cases and controls under 5 years of age by health facilities located in either peri-urban or urban settings in Blantyre, Malawi, sampled between April 2012 and October 2015

Health facility	Studied		Enrolled		
	Cases, N (%)	Controls, N (%)	Total, N (%)	Cases, N (%)	Control, N (%)
Peri-urban					
*Mpemba*	46 (25)	71 (25)	482 (15)	139 (28)	343 (13)
*Chileka*	68 (36)	89 (31)	549 (17)	186 (38)	363 (14)
Sub-total	114 (61)	160 (56)	1031 (33)	325 (66)	706 (27)
Urban					
*Zingwangwa*	19 (10)	33 (12)	584 (19)	44 (9)	540 (20)
*Bangwe*	15 (8)	24 (8)	522 (17)	33 (7)	489 (18)
*Chilomoni*	18 (10)	30 (10)	466 (15)	45 (9)	421 (16)
*Ndirande*	21 (11)	39 (14)	553 (18)	46 (9)	507 (19)
Sub-total	73 (39)	126 (44)	2125 (67)	168 (34)	1957 (73)
Overall total	187 (100)	286 (100)	3155 (100)	493 (100)	2663 (100)

### Risk factors in urban and peri-urban health facilities

When evaluating socio-economic and housing risk factors, higher level of education, electricity in the house and possession of TV were all protective against malaria (Table 2), with similar associations in both urban and peri-urban zones (P values for interaction of all these >0.05). Also protective against malaria, in both urban and peri-urban settings, were presence of piped water in the house and house completion (finished roof, floor, and wall). Having travelled and slept away in the month before contracting malaria was positively associated with malaria for participants overall (OR = 2.35; 95% CI = 1.04, 5.3), however, such travel was a strong risk for participants who visited urban HFs (OR among urban = 5.1; 95% CI = 1.62, 15.8; P value for interaction travel-urban = 0.03).Use of an LLIN the previous night was not associated with malaria (Tables 2, 3).

**Table 2:** Risk factors (crude odds ratios) for clinical malaria (cases) compared with age- and location-matched non-malaria controls among children under-5 years of age from urban and peri-urban health facilities of Blantyre city, Malawi, sampled between April 2012 and October 2015

Characteristic	Cases (N=187), n (%)	Controls (N=286), n (%)	OR (95% CI)	P value^‡^
Gender (male)	98 (52)	147 (51)	0.99 (0.67–1.47)	0.47
LLIN use previous night	157 (77)	233 (82)	0.75 (0.40, −1.2)	0.31
Slept away from house	18 (9)	15 (4)	2.35 (1.04–5.32)	0.04
Tertiary education	46 (25)	107 (38)	0.53 (0.34, 0.83)	0.002
Ownership of house	151 (53)	105 (57)	0.86 (0.55, 1.35)	0.51
Toilet in house	85 (3)	8 (3)	1.0 (0.32, 3.10)	1.0
Piped water in house	70 (38)	141 (50)	0.50 (0.26, 0.98)	0.04
Finished house roof	107 (58)	203 (73)	0.44 (0.27, 0.73)	0.001
Finished house floor	77 (42)	165 (58)	0.41 (0.25, 0.66)	0.0003
Finished house walls	119 (65)	212 (75)	0.58 (0.38, 0.90)	0.01
Electricity at house	26 (14)	72 (25)	0.44 (0.25–0.78)	0.005
TV ownership	20 (11)	69 (24)	0.39 (0.22, 0.69)	0.001
Radio ownership	90 (49)	146 (52)	0.89 (0.58–1.35)	0.57
Bike ownership	34 (19)	48 (17)	1.12 (0.67–1.86)	0.66
Car ownership	1 (0.6)	4 (1.4)	0.43 (0.05, 3.87)	0.45

*OR* odds ratio, *CI* confidence interval

^‡^P values, OR and 95% CI were estimated through conditional logistic regression accounting for matching

**Table 3:** Association between house finishing/facilities/ownership continuous scores and clinical malaria among children under 5 years of age in Blantyre, Malawi, sampled between April 2012 and October 2015

Score estimated by PCA analysis*	Overall OR (95% CI)	Stratified OR (95% CI)		OR Diff. P value Peri-urban vs. urban^‡^
		Peri-urban	Urban	
House finishing/piped water (PC1)	0.78 (0.63, 0.91)	0.74 (0.60, 0.91)	0.76 (0.55, 1.06)	0.87
House ownership/finished walls (PC2)	0.84 (0.68, 1.06)	0.87 (0.65, 1.17)	0.53 (0.56, 1.14)	0.72
Toilet in house (PC3)	1.09 (0.89, 1.32)	0.93 (0.70, 1.22)	1.36 (0.96, 1.92)	0.09

*OR* odds ratio, *CI* confidence interval, *PC* principal components

* Scores represent the first three principal components that explained 77% of the variability in the data. Scores were named according to loadings of 0.40 or larger

^‡^P values for interaction tests between each risk factor and urbanicity (vs. rural), OR and 95% CI were estimated through conditional logistic regression accounting for matching

### Score summarizing house ownership, finishing, and sanitation

To be able to analyse simultaneously variables related to house ownership, finishing, and sanitation in regressions, a PCA was performed (see Additional file 1: Table S1). The first three components explained most of the variability (~77%) in the data, with the first component particularly representing finished floor (floor with cement or tiles) and roof (roof with iron sheets) and presence of finished floor and roof. The second component primarily represented finished walls and ownership of the house, and the third component was associated with having a flush toilet at the house. Only the first component was associated with clinical malaria (OR = 0.78; 95% CI = 0.63, 0.91) both in the urban and peri-urban settings (P = 0.87) (Table 3).

### Multiple regression analysis for risk of clinical malaria

In multiple regression models, results of adjusted analyses were comparable to the crude analyses above. Electricity and higher-level education were negatively associated with odds of clinical disease, while travelling increased the odds, but only in the urban setting (Table 4). However, after accounting for the effect of the first principal component, only travel remained statistically significantly associated with clinical malaria in the urban setting, probably because of the high association between all socio-economic markers.

**Table 4:** Risk factors (adjusted odds ratios) for clinical malaria (cases) compared with age- and location-matched non-malaria (controls) among children under 5 years of age from urban and peri-urban health facilities of Blantyre city, Malawi, sampled between April 2012 and October 2015

Characteristic	OR (N_cases_ = 175, N_Controls_ = 275)	95% CI	P value^‡^
*Model 1*			
Slept away from home; *n (%)*			0.02**
In the urban* setting	2.36	(1.31, 4.2)	
In the peri-urban* setting	1.17	(0.64, 2.15)	
Household with electricity	0.57	(0.31, 1.06)	0.08
Tertiary education	0.59	(0.36, 0.96)	0.03
*Model 2*			
PC 1 (House finishing/ piped water score)	0.80	(0.66, 0.97)	0.02
Slept away from home; *n (%)*			0.04**
In the urban* setting	2.3	(1.28, 4.11)	
In the peri-urban* setting	1.19	(0.65, 2.20)	
House with electricity	0.76	(0.39, 1.47)	0.41
Tertiary education	0.67	(0.41, 1.12)	0.12

*OR* odds ratio, *CI* confidence interval

* Urban areas considered Bangwe, Ndirande, Zingwangwa and Chilomoni HFs; peri-urban areas are Chileka and Mpemba HFs

** P value for the interaction between urban vs. peri-urban and slept away from home

^‡^P values, OR, and 95% CI were estimated through conditional logistic regression to account for matching strata

### Entomological monitoring

In total, 35 female *Anopheles* mosquitoes were collected from the 473 study households, all from households of participants who attended peri-urban HFs. Thirty-two (91.4%) of the total *Anopheles* caught were identified as *Anopheles funestus* s.s. by PCR and the remainder were *Anopheles arabiensis*. None of the twenty-seven *Anopheles* sp included in the ELISA test for *P. falciparum* sporozoite detection came out positive. Study households were more likely to have ≥one *Anopheles* mosquito in peri-urban than in urban settings for both case (Fisher’s exact test = 0.012; P value < 0.05) and control households (Fisher’s exact test = 0.036; P value < 0.05). Considering only households (HH) from peri-urban HFs, more case HH (nine of 114) had ≥ one *Anopheles* mosquito than did control HH (six of 160) (Fisher’s exact test = 0.018; P value < 0.05). Although there was no *Anopheles* captured in the urban HH, more case HHs had ≥ one *Culex* mosquito (77%) than did the control HHs (56%) (P value < 0.05).

## Discussion

This case–control study in Blantyre, Malawi showed that travel and sleeping away from home was strongly associated with malaria illness for children aged 6–59 months attending urban HFs as compared to those seen at peri-urban HFs. This finding is similar to results from previous reports from this setting [6] and is consistent with studies from other major SSA cities where malaria is a public health problem [10–12]. Although travel has been shown to be a risk factor for urban dwellers in various investigations, our study showed that in the peri-urban area of Blantyre, travel was not the main predictive factor for malaria illness. This, coupled with the fact that all *Anopheles* mosquitoes in our study were collected from households of participants attending peri-urban HFs, suggests that active transmission occurs more often in the outskirts of Blantyre city. Although travel is strongly associated with malaria illness in the 6–59 months age group in Blantyre city, the effect of travel has not been quantified for other mobile demographic groups. Studies have shown that levels of population movement and individual infection rates differ among demographic groups [13, 14]. In Kenya and Tanzania, for example, the 20–30 year old group and female urban migrants appear to travel more, thereby transporting parasites from one place to another [14]. The nature of the travel (short-term vs. periodic/permanent) also is likely to affect behaviours related to malaria prevention, and therefore patterns of infection and movement of parasites between locations and sub-population groups [15]. Travel destinations were identified, but no efforts were made to assess whether travel was a labour-related, seasonal movement, short-term family visits, or permanent movement from rural to urban areas. In the case of Blantyre city, identifying and understanding these key mobile demographic groups, their behaviours and the pattern of parasite dispersal is critical to the design of more targeted interventions. Such interventions could include targeted prophylaxis or health education for high-risk groups travelling to rural hotspots for short-term family visits, school or farming.

However, travel is not the only reason for malaria illness: nearly 80% of the children with malaria illness in this study had not travelled outside the city limits in the month preceding the interview. This suggests that many of these children acquired infection inside the city limits. To demonstrate that transmission occurs in the urban setting, there is need to: (1) confirm that urban residents have *Plasmodium* infection but lack a history of recent travel; (2) document the presence of blood-fed adult *Anopheles* mosquitoes in/near urban residences; and (3) identify presence of peri-domestic breeding site near households [16]. In our study, attempts to capture by aspiration indoor-resting mosquitoes were successful for *Culex* species in both urban and peri-urban settings, but the only *Anopheles* adults captured were from households of participants attending peri-urban HFs. The absence of *Anopheles* mosquitoes in samples from households within urban Blantyre has previously been reported [17] and is in contrast to entomological findings from other cities in Africa where *Anopheles* mosquitoes have been identified [18–20]. It is not clear whether the scarcity of *Anopheles* sampled mosquitoes within Blantyre city limits is due to the genuine paucity of vectors in the city or the method of collection (the Prokopack aspirator). Blantyre city and district is located at high altitude with a resulting low seasonal transmission. The high altitude could explain the low density of vectors compared to other cities with more conducive environment for mosquito breeding [18–20]. The methods used for mosquito collection could also be the reason for low numbers collected. Although mosquito aspiration has been previously evaluated and validated [21], human landing catches are the commonly used and most sensitive method for monitoring mosquitoes in areas where the mosquito population is low [22] and most urban malaria studies have sampled mosquitoes using this approach [18–20]. However, the presence of *An. funestus* s.s. and *An. arabiensis* in peri-urban areas, provides partial evidence of potential local malaria transmission in areas surrounding Blantyre city as both species are known malaria vectors in Malawi [23]. Further research is needed though in Blantyre city to better characterize malaria vector abundance, feeding behaviour and breeding sites, as these represent factors important to transmission within the city limits.

The study showed that higher level of education for caregivers, possession of a TV, electricity and/or piped water in the house, and house finishing (iron sheet roof, cemented floor, brick wall) were all associated with protection from malaria illness, both in the urban and peri-urban zones. House finishing remained a significant protective factor even after controlling for other factors. Similar findings have been demonstrated in more remote, rural areas of Malawi where simple house improvements have been associated with lower risk of malaria [24]. These findings serve to highlight that malaria remains a disease of the poor [25] and that increased investment in socio-economic development should be one of the key interventions to reduce malaria [26], especially in areas like Malawi where the impact of malaria control has remained limited [27, 28].

Although previous research has shown that LLIN use is protective against malaria in this setting [6, 7], LLIN use was not associated with reduced risk of malaria in the present study. LLIN use for both cases (77%) and controls (82%) was high in the area following the national-wide mass LLIN distribution in 2013 where one LLIN was given to every two people. This lack of protective effect could therefore be due to community-wide protective effects of LLINs, as research has shown that in areas of high net use, LLINs confer protection on community members who may not even sleep under a net [29]. Since travel is also associated with malaria illness in this study, it is possible that LLINs were not used during travel, hence decreasing their effectiveness in preventing malaria illness. The lack of effectiveness also raises the possibility that insecticide resistance, which has been well documented in Malawi [30–32], could be contributing. More research on the role of insecticide resistance on LLIN effectiveness in the urban and peri-urban setting will help address this issue.

Among the limitations of this study is the fact that the analysis of risk factors is based on urban/peri-urban classification based on boundaries specified by the municipal government. Of concern here is the fact that within the urban city limit boundary, there is spatial heterogeneity of conditions with some areas resembling rural areas, especially in the poor but rapidly developing neighbourhoods where idle land is exploited for farming and other commercial activities. Our analysis fails to control for that heterogeneity. Despite this, travel as a risk factor was more pronounced in participants who were enrolled at urban facilities. Another limitation is the fact that case–control studies, as compared with longitudinal studies, are more prone to bias (selection, recall) and mistaken inference. To limit selection bias, cases and controls were matched from the same HF, zones and subjected to both diagnostic tests of microscopy and PCR. However, recall bias cannot be ruled out as travel history was over a period of 1 month, opening up a possibility that those with malaria could recall their travel histories more than those without malaria. Another limitation was that LLIN use was reported rather than observed, since the visits were made only during the day.

## Conclusion

This study has demonstrated that malaria in urban areas (but not peri-urban) is strongly associated with travel to rural areas, highlighting the need for interventions that would target the travelling public. However, detailed spatial and temporal information is required to understand the vector species, their breeding sites, and determinants of hotspots for transmission in these urban settings. Also it will be important to understand relevant behaviours of the groups at risk of malaria, in order to mount more effective urban malaria control and prevention programmes.

## Additional file

**Additional file 1: Table S1.** Weights (loadings) of household characteristics in each principal component.
