# Supplementary material for: Patterns and determinants of malaria risk in urban and peri-urban areas of Blantyre, Malawi
Source: Malar J. 2016 Dec 8;15:590. doi: 10.1186/s12936-016-1623-9 (PMC5146950; doi:10.1186/s12936-016-1623-9)
Supplement: Supplementary file 1 — Additional file 1: Table S1. Weights (loadings) of household characteristics in each principal component. [file 12936_2016_1623_MOESM1_ESM.docx]

**Additional File 1 – Weights (loadings) of household characteristics in each principal component.**

|  | **Principal components** | | | | | |
| --- | --- | --- | --- | --- | --- | --- |
|  | **1** | **2** | **3** | **4** | **5** | **6** |
| **Cumulative % of Explained Variability** | **(44%)** | **(61%)** | **(77%)** | **(88%)** | **(95%)** | **(100%)** |
| **Household characteristic:** |  |  |  |  |  |  |
| House ownership | -0.39 | 0.46 | 0.07 | 0.79 | 0.04 | 0.07 |
| Finished house floor | 0.52 | 0.002 | -0.02 | 0.33 | -0.54 | -0.57 |
| Finished house roof | 0.52 | 0.14 | -0.19 | 0.13 | -0.23 | 0.77 |
| Finished house walls | 0.30 | 0.71 | -0.34 | 0.23 | 0.42 | -0.25 |
| Piped water | 0.44 | -0.38 | 0.22 | 0.38 | 0.68 | -0.03 |
| Toilet in the house | 0.16 | 0.36 | 0.89 | -0.21 | -0.07 | 0.06 |
